# A bio-inspired approach for the design of a multifunctional robotic end-effector customized for automated maintenance of a reconfigurable vibrating screen

**DOI:** 10.1186/s40638-017-0060-8

**Published:** 2017-08-31

**Authors:** O. A. Makinde, K. Mpofu, R. Vrabic, B. I. Ramatsetse

**Affiliations:** 1 Department of Industrial Engineering, Tshwane University of Technology, Statsartillirie Road, Pretoria West, South Africa; 2 Manufacturing Engineering, University of Ljubljana, Ljubljana, Slovenia

**Keywords:** RVS machine, Robotic-driven maintenance, Robotic end-effector, Therblig, Morphology

## Abstract

The development of a robotic-driven maintenance solution capable of automatically maintaining reconfigurable vibrating screen (RVS) machine when utilized in dangerous and hazardous underground mining environment has called for the design of a multifunctional robotic end-effector capable of carrying out all the maintenance tasks on the RVS machine. In view of this, the paper presents a bio-inspired approach which unfolds the design of a novel multifunctional robotic end-effector embedded with mechanical and control mechanisms capable of automatically maintaining the RVS machine. To achieve this, therblig and morphological methodologies (which classifies the motions as well as the actions required by the robotic end-effector in carrying out RVS machine maintenance tasks), obtained from a detailed analogy of how human being (i.e. a machine maintenance manager) will carry out different maintenance tasks on the RVS machine, were used to obtain the maintenance objective functions or goals of the multifunctional robotic end-effector as well as the maintenance activity constraints of the RVS machine that must be adhered to by the multifunctional robotic end-effector during the machine maintenance. The results of the therblig and morphological analyses of five (5) different maintenance tasks capture and classify one hundred and thirty-four (134) repetitive motions and fifty-four (54) functions required in automating the maintenance tasks of the RVS machine. Based on these findings, a worm–gear mechanism embedded with fingers extruded with a hexagonal shaped heads capable of carrying out the “gripping and ungrasping” and “loosening and bolting” functions of the robotic end-effector and an electric cylinder actuator module capable of carrying out “unpinning and hammering” functions of the robotic end-effector were integrated together to produce the customized multifunctional robotic end-effector capable of automatically maintaining the RVS machine. The axial forces ($F_{{1{\text{t}}}}$ and $F_{{2{\text{t}}}}$), normal forces ($F_{\text{n}}$) and total load $(F_{\text{d}} )$ acting on the teeth of the worm–gear module of the multifunctional robotic end-effector during the gripping of worn-out or new RVS machine subsystems, which are 978.547, 1245.06 and 1016.406 N, respectively, were satisfactory. The nominal bending and torsional stresses acting on the shoulder of the socket module of the multifunctional robotic end-effector during the loosing and tightening of bolts, which are 1450.72 and 179.523 MPa, respectively, were satisfactory. The hammering and unpinning forces utilized by the electric cylinder actuator module of the multifunctional robotic end-effector during the unpinning and hammering of screen panel pins out of and into the screen panels were satisfactory.

## Background

RVS machine is a customized, flexible beneficiation machine capable of screening varying run-off mineral sizes and volumes in surface and underground mines based on the customers demand though rapid adjustment or geometric transformation of the screen structure and screen panel modules of the machine [1].

To this effect, the RVS machine can be configured from its initial configuration with a capability size of 2500 mm × 1500 mm to other configurations and 3000 mm × 1800 mm, 3500 mm × 2000 mm, 4000 mm × 2000 mm and 4700 mm × 2500 mm based on the machine maintenance manager in order to meet new customers requirements or recover production loss that emanate during the RVS machine maintenance [2].

In order to ensure safe and danger-free maintenance of this machine when utilized in dangerous and hazardous underground environments that are characterized with highly poisonous fumes and gases, unstable stopes and supports (i.e. flying rocks) and high amount of poisonous dust fumes that could result in lung diseases such pneumoconiosis and silicosis [3–5], accidents and deaths [6–9], there is a need to develop safer maintenance solutions and practices that would eliminate or reduce the exposure of maintenance managers of this machine to this dangerous/hazardous working environment as emphasized by Lirong et al. [10]. In view of this, robotic-driven maintenance solution capable of automatically, autonomously and telescopically (i.e. by means of a controller) carrying out the maintenance of this machine under little or no human supervision was proposed by Farnsworth et al. [11] and Makinde et al. [12] as a potential danger-free and safe maintenance philosophy for maintaining machines (which in this case was RVS machine) used in underground hazardous environment. However, to ensure the functionality of this robotic-driven maintenance system, a robotic end-effector which is capable of automatically carrying out all the varying maintenance tasks on the RVS machine is required by this robotic-driven maintenance system. In view of this, this paper proposes a bio-inspired system approach which unfolds a multifunctional robotic end-effector design capable of automatically maintaining the RVS machines when used in hazardous underground mine. In order to achieve this milestone, the second section of this paper reviews the bio-inspired system approaches utilized by researchers in designing robotic end-effector systems, the third section presents the approach or framework used for the design of the multifunctional end-effector capable of automatically maintaining the RVS machine, the fourth section presents a system observation study of how different RVS machine subsystems are maintained by maintenance manager, which thus unfolds the therblig and morphological methodologies that could serve as a guideline to meet the goals and or objective functions of the robotic end-effector, while the last section of this paper presents a multifunctional robotic end-effector solution capable of automatically maintaining the RVS machine (i.e. meeting the maintenance operations constraint and the objective functions of the RVS machine maintenance process).

## State of the art in the application of bio-inspired system in the design of robotic end-effectors

Different design approaches have been adopted by previous researchers for designing robotic end-effectors. Bralla [13], Pahl et al. [14], Krumenauer et al. [15] and Furtado et al. [16] provided an overview of design approaches, which ranges from design for excellence (DFX), design to weight (DTW), design for manufacturability (DFM) and design for reliability (DFR), that could be used in designing robotic end-effectors. They also emphasized that different concept alternatives capable of achieving the aforementioned design strategies need to be developed and evaluated using different decision techniques such as quality function development (QFD) and house of quality (HoQ) [17], weighted decision matrix (WDM) [18], analytical hierarchy process (AHP) [19] and analytical network process (ANP) [20]. However, this exercise required in ascertaining the best concept capable of meeting the aforementioned design strategies based on system design criteria and objectives are time-consuming, energy sapping and strenuous. To this effect, the adoption of a bio-inspired approach which emulates and mimics nature for the design of a system serves as a potential approach in reducing the time to design a robotic end-effector [21]. Recently, biologically inspired systems and control methods have been studied widely, in particular in robotics field. Thus, a number of virtual human or animal-like robots and control approaches have unfolded for the last decade. To be precise, bio-inspired systems are those systems where biology plays a vital part in solving problems in another domain [22]. By definition, bio-inspired design uses analogies of biological systems to develop solutions for engineering problems. Bar-Cohen [23] also defined bio-inspired robotics as medium of learning from nature and extremely complex and efficient biological systems, and mimicking similar mechanisms in order to solve a specific problem in the engineering field. The benefits from the bio-inspired systems in the area of robotics can be seen in several applications, including manipulator design [24], autonomous robotic system design, end-effector design, robotic vision system design [25–27] and control system design. Due to drawbacks caused by common traditional systems, many researchers have proposed new methodologies, strategies and approach such bio-inspired systems that could be used to automate the maintenance tasks in the industry [28]. Also in order to ensure firm gripping of different objects, advanced solutions such as anthropomorphic-driven solutions [29, 30] which exhibits high number of fingers and actuators capable of ensuring high degree of freedom of this end-effector similar to that of a human hand have been designed in the literature. Pillearachechige et al. [31] proposed a bio-inspired multitasking robotic gripper design which uses a bird-like claw mechanism that allows amphibious motion for walking, swimming and grasping on land. In addition, the viability of the claw mechanism for grasping objects can be compared to similar grasping capabilities of a human hand. Lotti et al. [32] from the University of Bologna described the design of a new anthropomorphic robotic hand.

This end-effector was designed flexibly to adapt many different end-effector configurations since it incorporates deformable elements such as joint hinges which allows it to open and close just like a human hand. Xu et al. [33] described the design and the development of a new anthropomorphic robotic finger which is made up of three (3) biomimetic joints which exhibits dynamic properties closer to that of human counterparts. The actuation of this robotic finger is achieved through a series of simplified antagonistic tendons whose joints and arms mimic similar movements and motions to that of a human hand. Ariyanto et al. [34] developed a low-cost, effective anthropomorphic robotic hand which is made up of six (6) joints and six (6) actuators capable of grasping objects, clicking the computer mouse as well as playing piano. Through the innovative mechanism of modified glove sensors, the robotic hand is capable of mimicking the motions performed by human being. Hanafrah et al. [35] conducted a study on designing an anthropomorphic robotic hand capable of mimicking human hand movements especially for power grip postures. This type of robotic end-effector is embedded with fingers that consist of three segments which are connected together by four joints amounting to a total of four (4) degrees of freedom (DOF). The structure of each of the fingers relatively follows actual hand dimension. Thumb can reach the upper base of the palm till the tip of other four fingers. To perform power grip, the palm of human hand acts as the main surface orientation to rely on for stabilization. Yeung et al. [36] designed a wrench system that utilizes a multi-finger configuration, which exhibits human-hand-like mechanism to handle parts of different geometries with different grasping points. This design was developed for robotic arms that are used for assembly of automotive body parts. The second-generation end-effector and robot work cell presented by Gurau and Amstrong-koch [37] was aimed at increasing the productivity of the automated assembly process and the capability of the robot to assemble larger-scale fuel cell stacks. The type of end-effector possesses a passive compliance system consisting of two linear block and rail mechanisms that compensate for the robot’s inherent limitations in accuracy, repeatability and lack of joint flexibility. Kordi, Husing and Corves [38] developed an adaptive robot end-effector capable of grasping, manipulating and laying out the textile material in the mould as well as a novel tool changer capable of changing the end-effector’s function to draping function, in order to drape the textile material in the mould. They achieved this design by understudying the relevant activities carried out by a human being in producing textile preforms. This end-effector consists of grasping points with mounted cryo-grippers. In addition to this, a drive unit situated at the centre of the gripper actuates a parallelogram mechanism that allows forming flexibility during operation. However, it could be inferred that most of the bio-inspired robotic end-effectors designed in the literature are capable of achieving a single work function such as gripping/grasping (i.e. pick and place), drilling, milling and plucking (i.e. harvesting) as well as auxiliary function such as inspection during product manufacturing. To this effect, the existing end-effectors are not equipped with all the functions required to carry out different maintenance tasks on the RVS machine. In light of this, the authors intend to develop a multifunctional robotic end-effector equipped with all the functions required to automatically maintain the RVS machine using the combination of the lesson learnt from the existing robotic end-effector approaches and new methodologies. The researcher adopted the aforementioned bio-inspired design approach discussed in this section to design a customized multifunctional robotic end-effector for RVS machine maintenance.

## Research approach

**Fig. 1:**
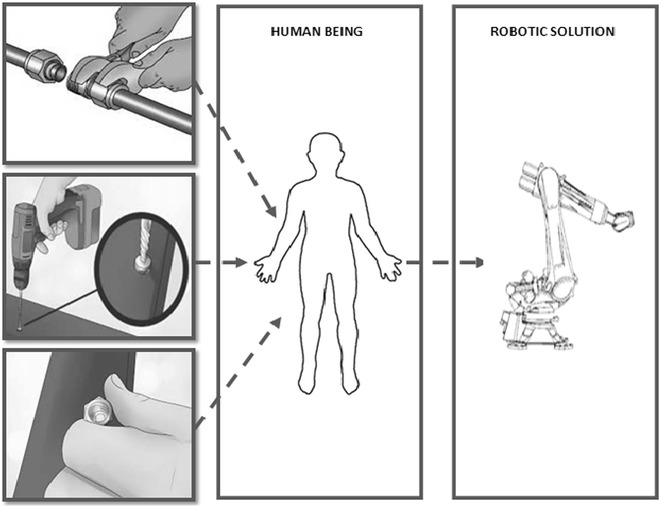
Analogy of maintenance tasks performed by a human: this image describes some of the maintenance activities performed manually by human being. This, on the one hand, includes the removal of tightened and loosened bolts using a spanner and a human hand and, on the other hand, portrays the tightening of bolts using a hand drill embedded with an Allen key module. In view of this, the image portrays how these human motions during the aforementioned activities can be automated using an intelligent robotic solution embedded with an end-effector capable of achieving these tasks

**Fig. 2:**
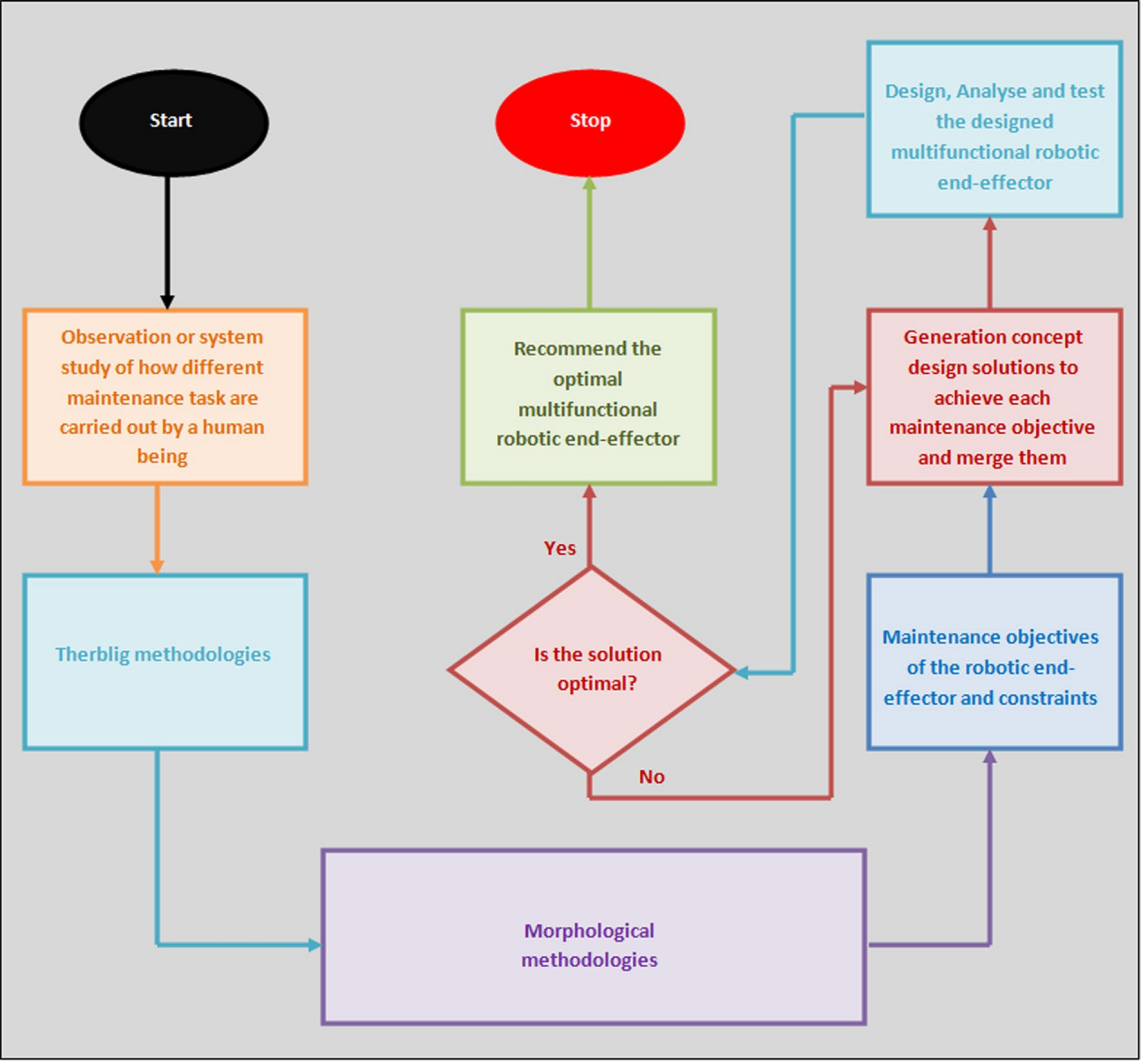
Research approach for development of multifunctional robotic end-effector: this image depicts the step-by-step procedures or activities carried out by the authors in designing the robotic end-effector. This entails the observation of how human being would carry out the RVS machine tasks, the analyses of the motions used by human being in carrying out the tasks and the different mechanisms required in automating these motions, which resulted in the modular design solution of the multifunctional robotic end-effector capable of automatically carrying out the RVS machine maintenance

We critically analysed the step-by-step procedures used by human being (i.e. maintenance managers) in maintaining the different subsystems of the RVS machine as shown in Fig. 1. Based on the result of the aforementioned exercise, we formulated the therbligs (i.e. capturing all the motions required in maintaining the different subsystems of the RVS machine through motion study) and morphological (i.e. functions) methodologies required to automatically maintain the RVS machine. Based on the result of the morphological assessment of the RVS maintenance tasks, we formulated the maintenance objective functions or goals of the robotic end-effector customized for maintaining the RVS machine. In light of this, we proposed a multifunctional robotic end-effector capable of achieving the maintenance objective functions or goals of the robotic end-effector as shown in Fig. 2 and, thereafter, validated its functionality through a detailed system design evaluation and simulation.

## RVS machine maintenance task study, therblig and morphological design approach

The possible and common frequent maintenance tasks that could be done on the RVS machine would include removal and replacement of worn-out RVS machine subsystems such as screen panels pin, screen panel, side plates, side liner plate, back plate, back liner plate, torsion bar, rosta suspensions, suspension brackets, M16 and M20 bolts and reconfiguration of the RVS machine in meeting the customer demands or recover production loss that could emanate during the maintenance of this machine [39].

### RVS machine maintenance task description

Detailed system observation of how human being is expected to carry out the aforementioned maintenance tasks is described extensively as follows:

1. Removal and replacement of worn-out pins and screen panel of RVS machine (*Task 1*).
2. Removal and replacement of worn-out side/back liner plates and back/side liner plates (*Task 2*).
3. Removal and replacement of suspension brackets (*Task 3*).
4. Removal and replacement of worn-out torsion bar of the RVS machine (*Task 4*).
5. Reconfiguration of the RVS machine along either the X or the Y direction or combination of both from initial configuration, 300 mm × 600 mm to 600 mm × 1200 mm (*Task 5*).

#### Task 1

- Loose using a pin tool removal, the worn-out pin(s) holding the screen panels to the screen deck frame of the RVS machine.
- Grip and transport the worn-out pin(s) into the waste bin where the worn-out RVS machine subsystems are disposed for recycling purposes.
- Grip and transport the loose worn-out screen panels on the RVS machine to the waste bin where worn-out RVS machine subsystems are disposed.
- Grip, transport and place new screen panels obtained from the ware house where new screen panels are stored on their exact locations on the RVS machine.
- Grip, transport and fit new screen panel pins on the exact location where they will hold firmly the screen panels into the deck frame of the RVS machine.
- Hammer in the screen pins into hollows of each screen panel in order to firmly join the screen panels into the screen deck frame of the RVS machine using hammering module of the pin tool removal.

The description of the maintenance tasks 2–5 is highlighted in Appendix 1. The RVS machine maintenance task case studies analysed in task 1 to task 5 holistically unfolded that the basic task functions required in maintaining the RVS machine involve the loosening and tightening of bolts, ungrasping and gripping of different sizes and shapes of the RVS machine subsystems, transportation of gripped worn-out or new RVS machine subsystems, placing of gripping worn-out or new RVS machine subsystems in the exact warehouse location and unpinning and hammering of screen panel pins during RVS machine by the active participation of the human arm, fingers of the hand, spanners and pin tool removal and hammer, respectively. Using the results of the RVS machine maintenance task study, the sets of motions used by the human being in carrying out these maintenance tasks are described in the next section of this paper.

### Therblig and morphological analysis of the RVS machine maintenance task description

**Fig. 3:**
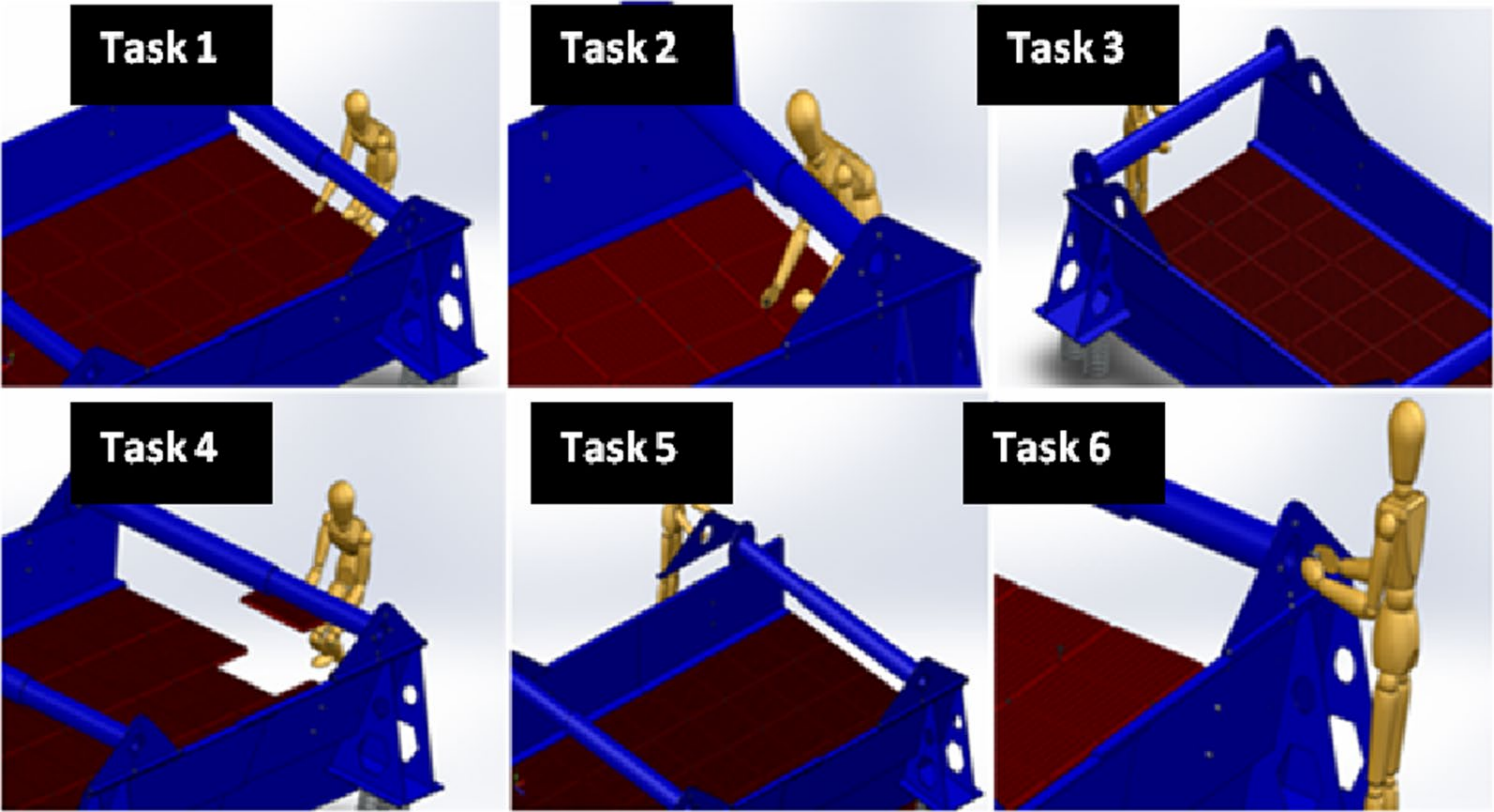
Analogy of maintenance tasks performed by a human: this image describes how a human being maintain the RVS machine by fitting the new screen panel on the screen deck, removing the screen panel pin on the surface of the screen during the maintenance of worn-out screen panels, removing worn-out torsion bar that needs to be replaced, gripping a new screen panel that needs to be fitted on the screen deck frame of the machine, gripping a new torsion bar bracket module that needs to be fitted to the torsion bar of the machine and screwing (pre-torque) a bolt using a human hand

**Fig. 4:**
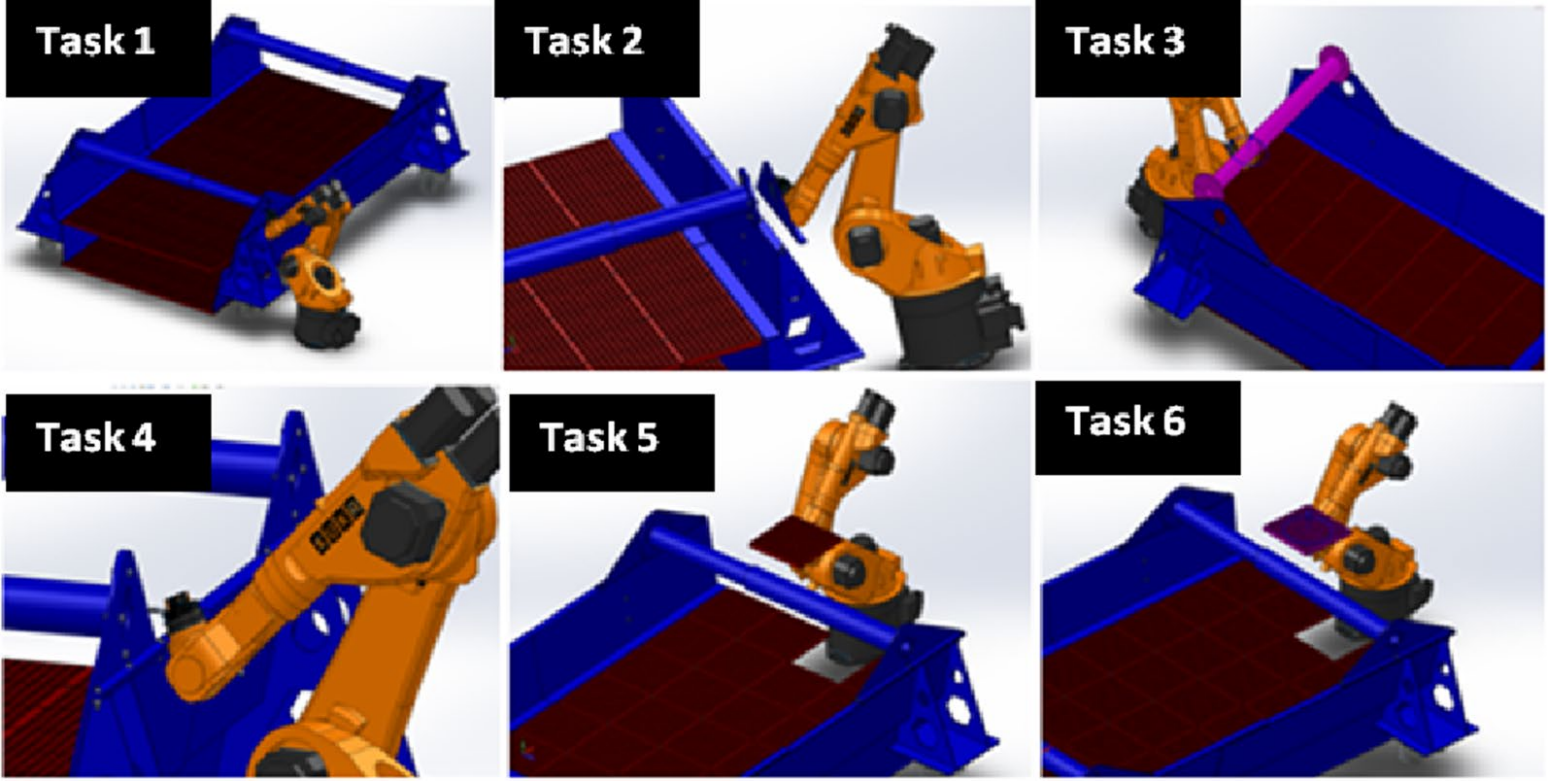
Analogy of maintenance tasks performed by a robot: this image describes how a robot maintains the RVS machine by screwing (torque) a bolt using a screw driving module of the robotic end-effector, grasping a new torsion bar bracket module that needs to be fitted to the torsion bar of the machine using the gripping module of the robotic end-effector, removing worn-out torsion bar that needs to be replaced using the gripping module of the robotic end-effector, loosening a bolt on the torsion bar bracket of the machine using the screw loosening module of the robotic end-effector and grasping a new screen panel that need to be fitted on the screen deck frame of the machine using the gripping module of the robotic end-effector

According to Jia et al. [40], therblig is a system used to assess and evaluate the different types of motions required in performing a task. In view of this, the sets of motions used by the maintenance managers of the RVS machine to carry out the maintenance task 1 to task 5 are illustrated in Table 1 as well as Appendix 2(a) to Appendix 5(a). In order to ascertain how these motions need to be exhibited using an automated system, a set of indices or parameters capable of automatically exhibiting these motions are presented in Table 2 as well as Appendix 2(b) to Appendix 5(b). In light of this, an analogy of how different RVS machine maintenance tasks are carried out using human being and an intelligent robotic solution is illustrated in Figs. 3 and 4. Based on these therblig results, a number of industrial engineering processes and motion information required in ascertaining the basic parameters required in automating these maintenance tasks, which are “unscrew”, “screw”, “ungrasp”, “grasp”, “transport loaded”, “position”, “release”, “unpin” and “hammer”, were mapped out, respectively. In light of this, the morphological results then holistically inferred that vision and recognition systems capable of mapping and identifying the positions of the object in an unknown environment, autonomous robot, equipped with manipulator, four-wheel drive system and a robotic end-effector capable of carrying out gripping, ungrasping functions, loosening and tightening functions as well as unpinning and hammering function, are required for automatically maintaining the reconfiguration of the RVS machine. Therefore, based on these analyses, it could be inferred that the maintenance objective functions (MOF) of the robotic end-effector (RE) (used by the robotic manipulator of the autonomous robot in carrying out the RVS machine maintenance tasks) are “gripping (G) and ungrasping (Ug)” functions (MOF 1), “loosening (L) and tightening (T)” functions (MOF2) and “unpinning (Up) and hammering (H)” functions (MOF3). An elaborated maintenance problem formulation for the robotic end-effector customized for automated maintenance of RVS machine is highlighted as follows:

**Table 1:**
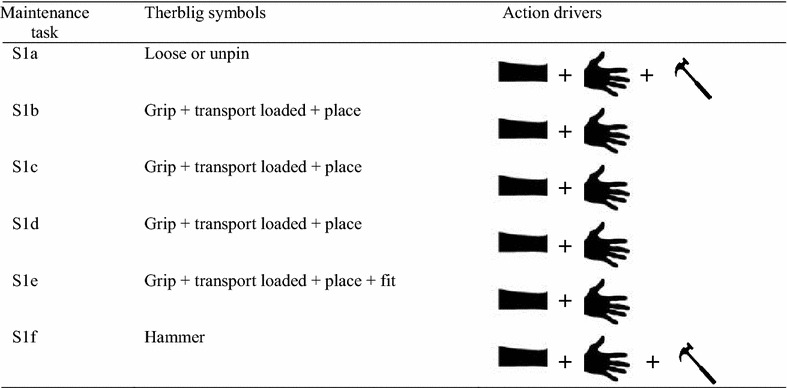
Therblig algorithm for task 1

**Table 2:**
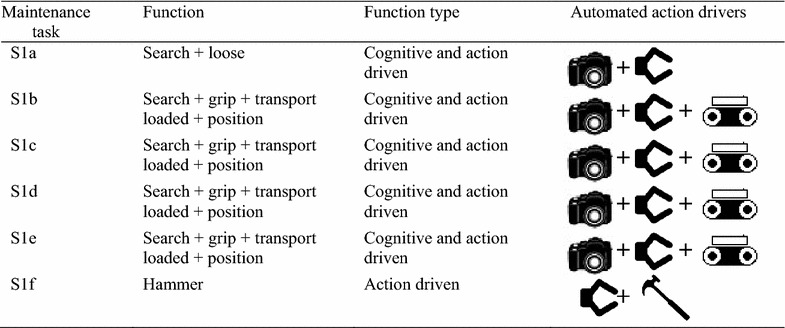
Morphological algorithm for task 1

Maximize the availability of RE: (capable of L and T, G and Ug, Up and H).

Subject to the removal of:

$${\text{SP}} \le 2$$

$${\text{IB}} \le 36$$

$${\text{EB}} \le 70$$

$${\text{BP}} = 1$$

$${\text{TB}} = 1$$

$${\text{SB}} \le 4$$

$${\text{RS}} \le 6$$

$${\text{SLP}} \le 2$$

$${\text{BLP}} = 1$$

$${\text{VM}} \le 2$$

$${\text{ScPa}} \le 8$$

$${\text{ScPaPi}} \le 24$$

$${\text{SP}},{\text{IB}},{\text{EB}},{\text{BP}},{\text{SD}},{\text{SB}},{\text{RS}},{\text{SLP}},{\text{BLP}},{\text{VM}},{\text{ScPa}},{\text{ScPaPi}} \ge 0$$

where SP is the “side plate”, IB is the “internal bolts”, EB is the “external bolts”, BP is the “back plate”, TB is the “torsion bar”, SB is the “suspension bracket”, RS is the “rosta suspension”, SLP is the “side liner plate”, BLP is the “back liner plate”, VM is the “vibrating motor”, ScPa is the “screen panel” and ScPaPi is the “screen panel pin” as shown in Fig. 5.

**Fig. 5:**
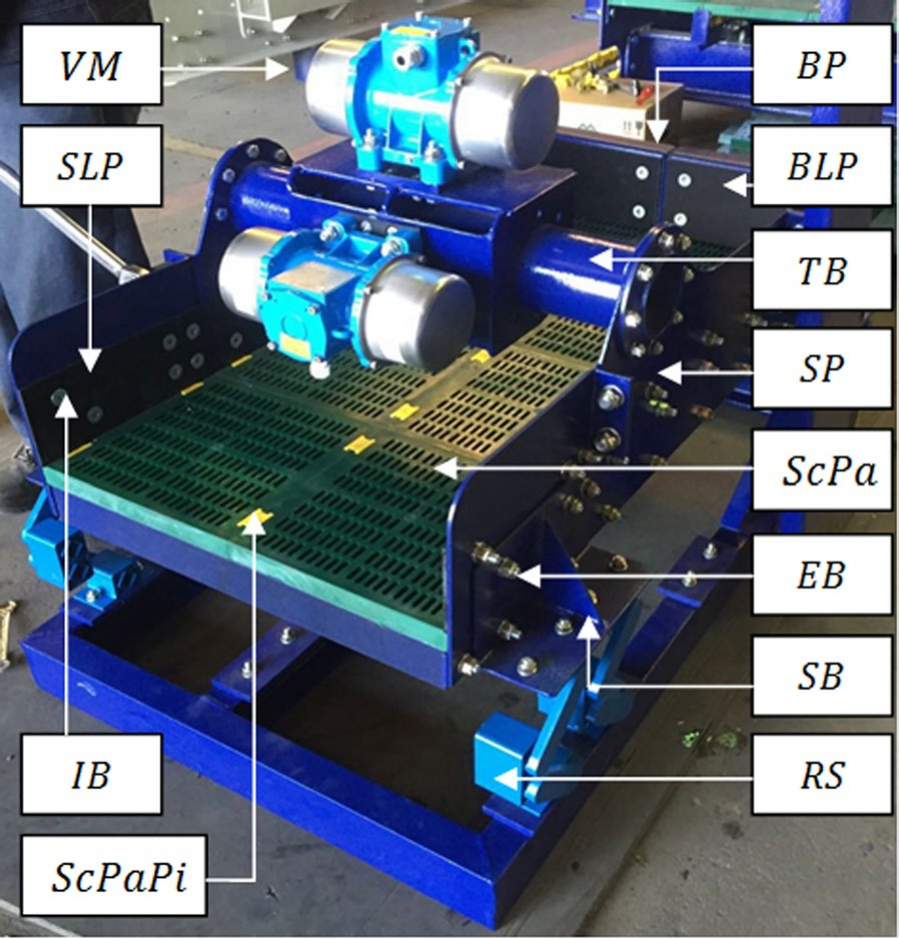
RVS machine prototype: this image portrays a functional RVS machine prototype that is made up of different components such as vibrating motor (VM), screen liner plate (SLP), internal bolts (IB), screen panel pin (ScPaPi), back plate (BP), back liner plate (BLP), torsion bar (TB), screen plate (SP), screen panel (ScPa), external bolt (EB), suspension bracket (SB) and rosta suspension (RS)

## Mechanism of multifunctional end-effector

**Fig. 6:**
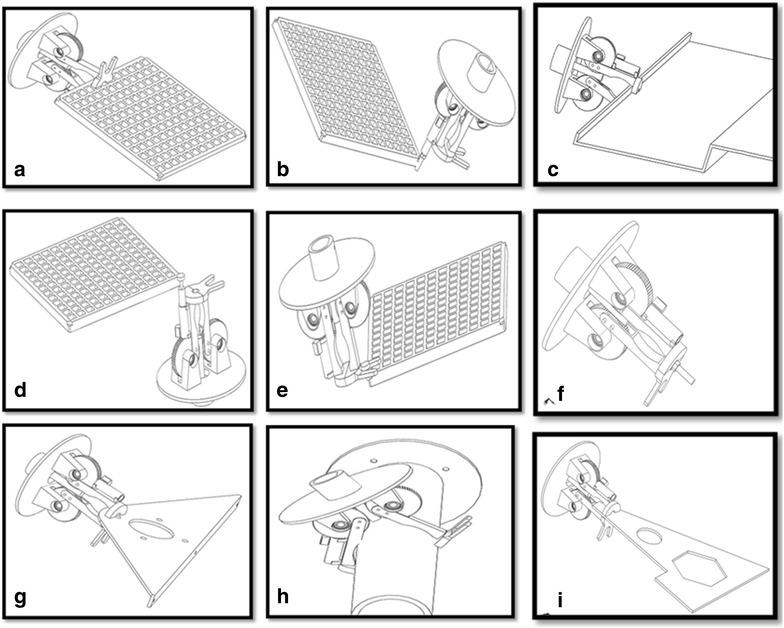
Maintenance tasks performed by the robotic end-effector on different RVS machine subsystems: this image showcases the different activities that the multifunctional end-effector is capable of carrying out the “gripping and ungrasping”, “loosening and bolting” and “unpinning and hammering” functions on the RVS machine. **a** Describes how the two rectangular fingers modules lap on each side of the screen panel in transporting it to and fro during the machine maintenance. **b** Explains the hitting actions performed by the multifunctional end-effector in hammering the screen panel pins into the screen deck frame using the electric actuation cylinder module. **c** Indicates how the two rectangular finger modules lap on each side of the side plate to transport it to and fro during the machine maintenance. **d** Demonstrates the hitting actions performed by the multifunctional end-effector in unpinning the screen panel pins from the screen deck frame using the electric actuation cylinder module. **e** Illustrates a fork-like bracket attached to one of the finger modules in gripping the loosened worn-out or new screen panel pins during the RVS machine maintenance. **f** Portrays the loosening and tightening of bolts holding or fastening two RVS machine subsystems together. **g** Articulates how the two rectangular finger modules lap on each side of the torsion bar bracket in transporting it to and fro during the machine maintenance. **h** Describes how the two rectangular finger modules of the robotic end-effector lap on a new or worn-out torsion bar during the RVS machine maintenance. **i** Unfolds how the two rectangular finger modules of the robotic end-effector lap on each side of the suspension bracket during the RVS machine maintenance

**Fig. 7:**
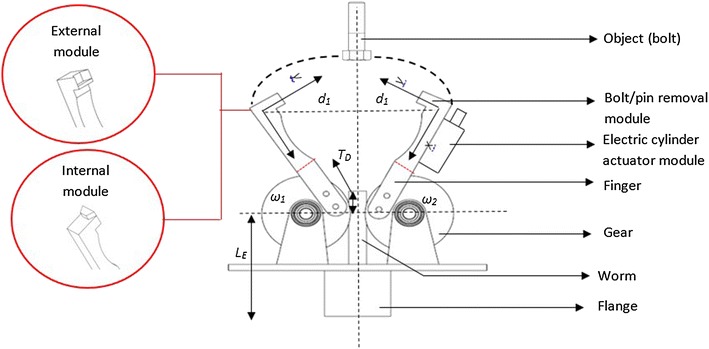
Schematic representation of the multifunctional robotic end-effector customized for maintaining the RVS machine: this image displays a schematic representation of different components of the multifunctional robotic end-effector. These components include socket-like module, electric cylinder actuator module and pin gripper module capable of achieving different maintenance tasks on the RVS machine. Furthermore, the image highlights the internal and external half-hexagonal shaped bracket modules that could be attached to the tip of the fingers of the robot end-effector

In order to achieve the objective function 1 or goal of the robotic end-effector (MOF1) customized for maintaining the RVS machine, two rectangular finger modules, which are endowed with the capability of lapping on each side of some RVS subsystems such as side plates, side liner plates, back plates, back liner plates, screen panels, screen panel pins, at the tip of each finger module as depicted in Fig. 6a, c, g, i to ensure these RVS machine subsystems’ firm gripping and are also designed to have a curve-shaped hollow compartment on the upper arm of each of the fingers as depicted in Fig. 6h in order to harbour or house cylindrical objects such as the worn-out or new torsion bar during their maintenance on the RVS machine, were considered by the authors as a suitable modular design element 1 capable of carrying out the gripping and ungrasping function. In addition to this, a set of plug-and-play internal and external socket-inspired brackets, (modular elements) made up of internal half-hexagonal-shaped brackets depicted in Fig. 7 attached to the tip of the finger modules in order to ensure the loosening and tightening of internal bolts fastening the side/back plates to the side/back liner plates as well as ensure the loosening and tightening of external bolts holding or fastening other RVS machine subsystems as depicted in Fig. 6f during RVS machine maintenance, were considered as modular design element 2 capable of achieving MOF2 of the robotic end-effector. An electric actuator module which can be obtained off-the-shelf, capable of unpinning of screen panel pin from the screen panels through the relative pull-out force applied on the lower tip of the screen panel pin as depicted in Fig. 6d, as well as hammering screen panel pins into the holes of screen panels and screen deck frame as depicted in Fig. 6b, in order to hold them together during the maintenance and configuration of the RVS machine is another suitable modular design element (i.e. module design element 3) capable of achieving MOF3 of the robotic end-effector. A fork-like bracket attached to one of the finger module is used for gripping the loosed worn-out or new screen panel pins during the machine maintenance as depicted in Fig. 6e. In order to meet all the maintenance objective functions or goals of the robotic end-effector, the modular design element 1, the modular design element 2 and modular design element 3 were then integrated together in order to produce a multifunctional robotic end-effector capable of automatically maintaining and reconfiguring the RVS machine when used in the mining industries.

**Fig. 8:**
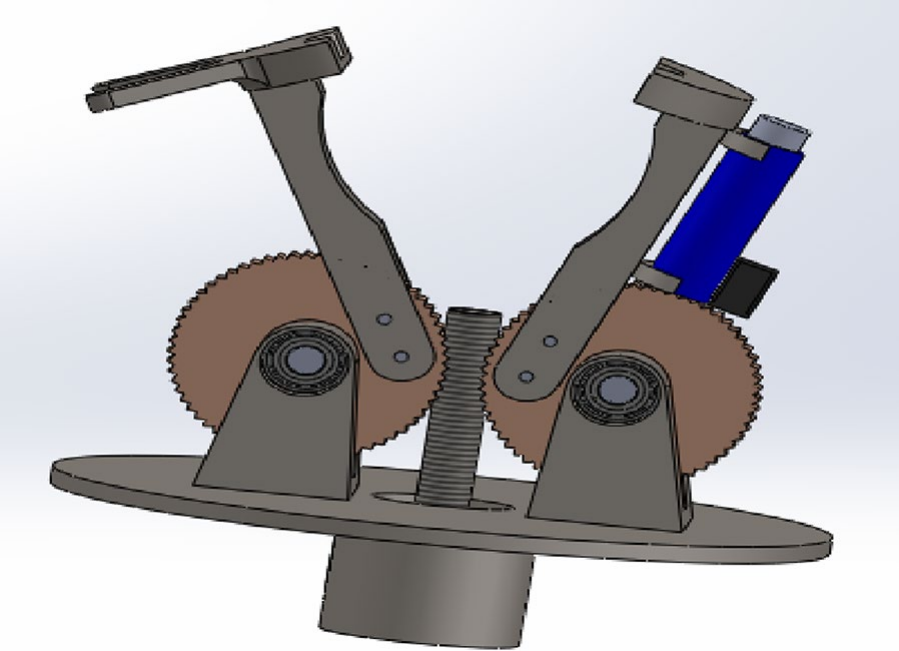
3D representation of the multifunctional robotic end-effector customized for maintaining the RVS machine: this image displays a three-dimensional CAD model of the multifunctional robotic end-effector embedded with modules such as finger modules, socket-like module, electric cylinder actuator module, pin gripper module and worm–gear module capable of carrying out different maintenance tasks on the RVS machine

In order to control the relative movements of fingers in gripping and ungrasping the RVS machine subsystems, two worm wheels attached on each side of the figures of the robotic end-effector which rolls on a gear powered by an electric motor to produce clockwise and anticlockwise rotation as depicted in Fig. 8 were considered by the authors as a suitable control system to achieve MOF 1 of the robotic end-effector, which are the inward and the outward movements of the fingers. In order to achieve the loosening and tightening of internal and external bolts by the internal and external socket-inspired brackets attached at the top of fingers of the robotic end-effector, the fingers of the robotic end-effector are driven inwards to lap on each other by the worm and gear module powered by the electric motor, after which the lapping fingers of the robotic end-effector are positioned on the internal or external bolts and then rotated by electric motor powering the wrist of the robotic manipulator to produce the clockwise and anticlockwise motions needed to torque or loose the bolts during the maintenance of the RVS machine. The relative pull-out or push-in forces applied on the screen panel pins through the pistons of the electric cylinder during the unpinning and hammering of these pins are powered by an electric motor which provides the clockwise motions required to drive the piston out of the cylinder in providing the pull-out and pushing forces as well as provide the anticlockwise motion required to drive in the piston of the cylinder after the unpinning and hammering actions of the screen panel pins during RVS machine maintenance.

## Design modelling and simulation of a multifunctional robotic end-effector for maintaining the RVS machine

### Worm and gear design analysis for the gripping and ungrasping actions of multifunctional robotic end-effector

In order to design an end-effector capable of gripping and ungrasping worn-out or new RVS machine subsystems using a worm and gear, a reverse engineering design approach, which involves: (1) presuming a worm and gear module using the worm and gear catalogue as a guideline, (2) determining the maximum power that will be dissipated during the gripping actions and (3) checking whether the presumed worm and gear module will not fail during these gripping actions through a detailed design, was carried out in this section.

**Fig. 9:**
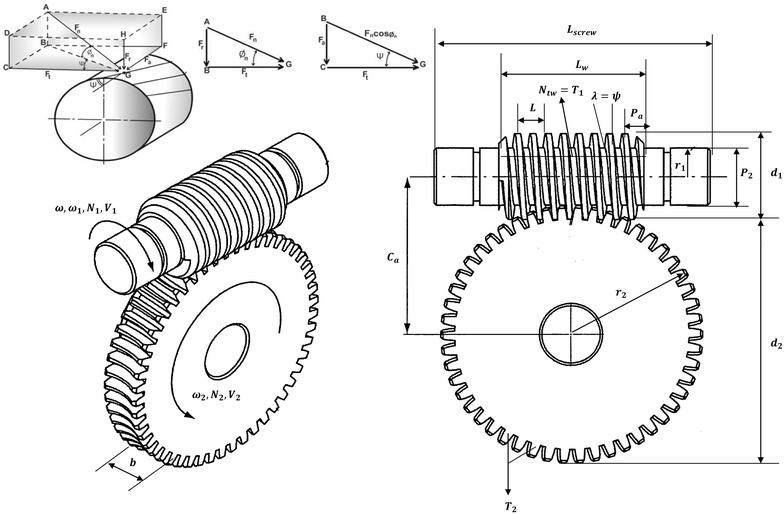
Schematic representation of the worm–gear module of the multifunctional robotic end-effector: this image displays all the parameters utilized during the design of the worm and gear module of the end-effector

To this effect, using the worm–gear catalogue as a guideline, a worm–gear module which has a worm, rotating at a speed of 150 rev/min with five (5) teeth and a wheel or gear of 20 teeth was presumed as the duplex worm wheel feature of the robotic end-effector. The descriptions of the parameters used in the system design, as well as their schematic representation, are shown in Table 3 and Fig. 9. In light of this, the gripping force and power generated by the robotic end-effector when gripping and ungrasping worn-out or new RVS machine subsystems such as screen panels, torsion bar, torsion bar bracket, side plate, side liner plates, back plate and back liner plate during RVS machine maintenance, obtained using Eqs. (1) to (4), are highlighted in Table 4.

1 $$F = \frac{\text{Mg}}{\mu } \times {\text{FS}}$$

2 $$\omega = \frac{2\pi n}{60}$$

3 $$V = \omega r$$

4 $$P = F \times V$$

Since the maximum power dissipated during the gripping and ungrasping of the RVS machine subsystems is 230.937 W (i.e. worst-case scenario) based on Table 4, then a worm–gear design, capable of delivering 230.937 W from the worm (i.e. lead screw or shaft) of the presumed off-the-shelf worm–gear, rotating at a speed of 150 rpm (i.e. *N*_1_) to the gear rotating at a speed *N*_2_ rpm in order to ensure firm gripping of different RVS subsystems by the robotic end-effector during RVS machine maintenance, was sought after by the authors.

**Table 3:** Parameters for the design of a multifunctional robotic end-effector

Notation	Description
*F*_g_	Gripping force of the multifunctional robotic end-effector
*M*	Mass of the object to be grasped by the multifunctional robotic end-effector
*g*	Acceleration due to gravity
*μ*	*μ* = coefficient of static friction between the object to be gripped and the multifunctional robotic end-effector =
FS	Factor of safety = 1.5
*ω* = *ω*_1_ and *ω*_2_	Relative angular speed of the worm and gear of the multifunctional robotic end-effector
*n*	Rotational speed of the worm of the multifunctional robotic end-effector
*V*	Linear speed of the worm of the multifunctional robotic end-effector
*r*	Radius of the worm of the multifunctional robotic end-effector
*P*	Power dissipated by the multifunctional robotic end-effector in carrying out a RVS machine maintenance task
*N*_1_ and *N*_2_	Rotational speed of the worm and gear of a multifunctional robotic end-effector
*T*_1_ and *T*_2_	Number of teeth in the worm and gear of a multifunctional robotic end-effector =
*i*	Worm–gear ratio
*C*	Assumed centre distance of the worm–gear of the multifunctional robotic end-effector
*d*_1_ and *d*_2_	Diameters of the worm and gear of the multifunctional robotic end-effector
*P*_2_	Circular pitch of the gear
*m*	Module
*P*_a_	Axial pitch of the worm
*L*	Lead of the worm
*N*_tw_	Number of the teeth on the worm
λ and Ψ	Lead angle and helix angle
*L*_w_	Axial length of the worm
*L*_screw_	Total length of the lead screw
*V*_1_ and *V*_2_	Linear speed of the worm and gear of the multifunctional robotic end-effector
*r*_1_ and *r*_2_	Radius of the worm and gear of the multifunctional robotic end-effector
*V*_s_	Sliding velocity
*F*_1t_, *F*_2t_, *F*_n_ and *F*_d_	Tangential forces, normal force and total force acting on the worm
*F*_a_ and *F*_r_	Thrust or axial force and radial force
${\o}_{\text{n}}$	Pressure angle
*F*_b_	Bending fatigue strength of the worm–gear of the end-effector
*F*_w_	The maximum allowable value of dynamic load under surface fatigue condition
*σ*_b_	Permissible bending stress in bending fatigue for worm–gear material
*b*	Face width
Kw	Material and geometry factor of the worm–gear
*Y*	Modified Lewis form factor obtained from a worm–gear catalogue
*ƞ*	Efficiency of the worm–gear module
*f*	Coefficient of friction acting on the worm–gear module
*H*_g_	Heat dissipation of the worm–gear
*A*	Surface area
*C*	The distance between the shafts of the duplex worm wheel of end-effector
*C*_H_	Heat transfer coefficient
*T*_o_	Lubricating oil temperature of the worm–gear
*T*_a_	Ambient air temperature
*σ*_a_ and *σ*_max_	Nominal and maximum bending stresses acting on the bolt socket module of the robotic end-effector
*τ*_a_ and *τ*_max_	Nominal and maximum torsional stresses acting on the bolt socket module of the robotic end-effector
Mc	Turning effect on the M-20 bolt
*T*	Torque on the bolt
*I*	Inertia of the object
*d*	Diameter of the bolt socket module
*K*_t_ and *K*_ts_	Theoretical stress concentration factors
$m_{{{\text{cyl-rod}}\;{\text{at}}\; 0\;{\text{str}}}}$	Mass of the cylinder rod at zeroth stroke
$m_{\text{cyl-rod}}$	Mass of the cylinder rod
$m_{\text{cyl-rod/mmstroke}}$	Mass of the cylinder rod per mm of stroke
$m_{\text{ext}}$	External mass = mass of the screen panel pin on the RVS machine
$F_{\text{fr-ext}}$	External frictional force in N
$F_{\text{ham}}$	Hammering force
$F_{\text{un-pin}}$	Unpinning force
$F_{\text{x,ext}}$	External force
$U_{\text{m}}$ and $V_{\text{m}}$	Initial and final linear speed of the electric motor powering the electric cylinder actuator
*a*	Acceleration of the electric motor
*s*	Travel distance of the piston of the electric cylinder actuator

**Table 4:** Calculated gripping force and power required by the multifunctional robotic end-effector during RVS machine maintenance

RVS machine subsystems	Weights (kg)	Gripping force (N)	Power (W)
Screen panels	3.60336	176.56	33.285
Torsion bar	13.42415	657.78	124
Torsion bar bracket	2.6075	127.77	24.087
Side plate	25	1225	230.937
Side liner plates	6.6	323.4	60.967
Back plate	12.4	607.6	114.545
Back liner plate	3.273	160.377	30.234
M-20 bolt	0.156	7.644	1.441

 The worm–gear ratio (i), diameters of the worm and gears (*d*_1_ and *d*_2_), circular pitch of the gear (*P*_2_), module (m), actual centre distance of the worm–gear module (*C*_a_), axial pitch of the worm (*P*_a_), lead of the worm (*L*), lead angle (λ), helix angle ($\Psi$), axial length of the worm (*L*_w_), total length of the lead screw (*L*_screw_) and linear speeds of the worm and gear (*V*_1_ and *V*_2_) of the worm–gear module of the robotic end-effector (depicted in Table 5) capable of delivering a maximum power of 230.937 W during the RVS machine maintenance were determined using Eqs. (5) to (17).

5 $$\frac{{N_{1} }}{{N_{2} }} = \frac{{T_{2} }}{{T_{1} }} = i$$

6 $$\frac{{C^{0.875} }}{3.0} \le d_{1} \le \frac{{C^{0.875} }}{1.7}$$

7 $$P_{2} = \frac{{d_{1} }}{5}$$

8 $$m = \frac{{P_{2} }}{\pi }$$

9 $$d_{2} = mT_{2}$$

10 $$C_{\text{a}} = 0.5 \left( {d_{1} + d_{2} } \right)$$

11 $$P_{\text{a}} = \frac{1.27m}{0.386}$$

12 $$L = N_{\text{tw}} \times P_{\text{a}}$$

13 $$\tan \lambda \;{\rm or}\;\Psi = \frac{L}{{\pi d_{1} }}$$

14 $$L_{\text{w}} = P_{\text{a}} \left( {4.5 + \frac{{T_{2} }}{50}} \right)$$

15 $$L_{\text{screw}} = {\text{length}}\;{\text{of}}\;{\text{the}}\;{\text{end}}\;{\text{effector}}\;{\text{hub}}\;(L_{\text{E}} ) + \left( {\frac{{{\text{axial}}\;{\text{length}}}}{2}} \right) + {\text{tolerance}}\;{\text{distance}}\left( {T_{\text{D}} } \right)$$

16 $$V_{1} = \omega_{1} r_{1}$$

17 $$V_{2} = \omega_{2} r_{2}$$

The friction acting on the worm–gear module of the robotic end-effector during RVS machine maintenance, obtained using the corresponding sliding velocity value, determined from Eq. (18) solution (which is *V*_s_ = 0.217 m/s) on the worm–gear catalogue is 0.064.

18 $$V_{\text{S}} = \frac{{V_{1} }}{\cos \lambda }$$

**Table 5:** Calculated design parameters of the worm–gear module of the multifunctional robotic end-effector

Design parameters	Designed values
Worm–gear ratio ($i$)	4
$d_{1}$ and $d_{2}$	24.50, 40 mm
$P_{2}$	4.90 mm
$C_{\text{a}}$	32.25 mm
$P_{\text{a}}$	6.58 mm
$m$	1.56–2 mm
$L$	32.90 mm
$\lambda$ or $\psi$	29.86°
$L_{\text{W}}$	32.24 mm
$L_{\text{Screw}}$	141.12 mm
$V_{1}$	0.18852 m/s
$V_{2}$	0.236 m/s

The tangential force ($F_{{1{\text{t}}}}$), the axial force ($F_{{2{\text{t}}}}$), normal force ($F_{\text{n}}$) and total force or load ($F_{\text{d}}$) acting on the teeth of the worm of the robot end-effector, which were determined using Eqs. (19)–(21), and the corresponding pressure angle (${\o}_{\text{n}}$) obtained from the worm–gear catalogue are highlighted in Table 6.

19 $$F_{{2{\text{t}}}} = F_{{1{\text{t}}}} = \frac{P}{{V_{2} }}$$

20 $$F_{\text{n}} = \frac{{F_{{2{\text{t}}}} }}{{\cos {\o}_{\text{n}} \cos \lambda }}$$

21 $$F_{\text{d}} = F_{{2{\text{t}}}} \left( { \frac{{6.1 + V_{2} }}{6.1}} \right)$$

**Table 6:** Results of the forces acting on the teeth of the worm–gear module of the multifunctional robotic end-effector during RVS machine maintenance

Forces acting on the teeth of the worm–gear module	Value (N)
$F_{{1{\text{t}}}}$	978.547
$F_{{2{\text{t}}}}$	978.547
$F_{\text{n}}$	1245.06
$F_{\text{d}}$	1016.406

 The bending fatigue strength of the worm–gear of the end-effector ($F_{\text{b}}$) and the maximum allowable value of dynamic load under surface fatigue condition ($F_{\text{w}}$) allowed to act on the worm–gear module of the robotic end-effector for its safe operation, which were determined using Eqs. (22)–(23), are highlighted in Table 7.

22 $$F_{\text{b}} = \left[ {\upsigma_{\text{b}} } \right] bmY$$

where $$b \le 0.5\left( {d_{1} + 2m} \right)$$

**Table 7:** Results of the bending and wear loads acting on the teeth of the worm–gear module of the multifunctional robotic end-effector during RVS machine maintenance

Bending and wear loads concentration on the teeth of the worm–gear module	Value (N)
$F_{\text{b}}$	1059.59
$F_{\text{w}}$	978.547

23 $$F_{\text{w}} = d_{2} bK_{\text{w}}$$

Since $F_{\text{b}}$(1059.52 N) > $F_{\text{d}}$ (1016.406 N) and $F_{\text{w}}$ (1043.28 N) > $F_{\text{d}}$ (1016.406 N), hence the design is safe from fatigue and wear rate/strength considerations.

The efficiency of the worm–gear module for the robotic end-effector (*η*), obtained using Eq. (24), is 0.8484 (84.84%).

24 $$\eta = \frac{{\cos {\o}_{\text{n}}-f\tan \lambda }}{{\cos {\o}_{\text{n}}-f\cot \lambda }}$$

The overall efficiency of a worm–gear module of the robotic end-effector during RVS machine maintenance was less than 100% (i.e. 84.84%) because of friction losses in the bearings and shaft seals and because of “churning” of the lubricating oil.

The time rate of heat dissipation of the worm–gear (*H*_g_), surface area (*A*) for the conventional housing designs of the worm–gear and the lubricating oil temperature of the worm–gear (*T*_o_) during RVS machine maintenance, obtained using Eqs. (25) to (27), are highlighted in Table 8.

25 $$H_{\text{g}} = \left( {1-\eta } \right) P$$

26 $$A = 14.75 C^{1.7}$$

27 $$H_{\text{g}} = C_{\text{H}} \left( {T_{\text{o}} {-}T_{\text{a}} } \right)$$

**Table 8:** Thermal capacity results acting on the worm–gear module during RVS machine maintenance

Temperature	Value
$H_{\text{g}}$	35.01 J/s
$A$	0.0298 m^2^
$T_{0}$	38.501 °C

Since the temperature of the lubricating oil of the worm–gear ($T_{\text{o}}$) is less than 93 °C (i.e. $T_{\text{o}}$ = 38.501 °C) for the permissible lubricating oil of the worm and gear (Shigley, 2011:765), hence the thermal design consideration of the worm–gear module of the robot end-effector is satisfactory.

### Socket-inspired bracket design analysis for loosing and tightening of bolts during RVS machine maintenance

**Fig. 10:**
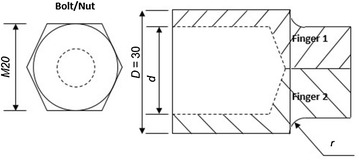
Schematic representation of the socket module design of the multifunctional robotic end-effector: this image displays a socket-like module of the multifunctional robotic end-effector embedded with hexagon internal feature required in removing and inserting bolts during RVS machine maintenance

In order to repair or replace worn-out subsystems of the RVS machine, the loosening and tightening of fasteners (i.e. M-20 bolts) and holding these subsystems in place need to be achieved. To achieve these, the two half-rounded hollow hexagonal machined metals, one positioned on the upper left bar of the robotic end-effector finger and the other positioned on the right-hand side bar of the robotic end-effector finger, that are inspired from a “bolt socket functional mechanism” as depicted in Fig. 10, were considered as a suitable design concept for the removal or tightening of the M-20 bolt.

Consider an M-20 bolt that is gripped by two half-rounded hollow hexagonal machined metals at tips of fingers of the robotic end-effector (i.e. a bolt socket module) as shown in Fig. 9, the nominal and maximum bending stresses ($\sigma_{\text{A}}$ and $\sigma_{ \rm{max} } )$ as well as the nominal and maximum torsional stresses ($\tau_{\text{A}}$ and $\tau_{ \rm{max} }$) acting on the bolt socket module of the robotic end-effector during the loosening and tightening of a bolt, which were obtained using Eqs. (28) to (31), are highlighted in Table 9.

28 $$\sigma_{\text{A}} = K_{\text{t}} \frac{\text{Mc}}{I}$$

29 $$\tau_{\text{A}} = \frac{{16K_{\text{ts}} T}}{{\pi d^{3} }}$$

30 $$\sigma_{ \rm{max} } = \frac{{\sigma_{\text{A}} }}{2} + \sqrt {\frac{{\sigma_{\text{A}} }}{2}^{2} + \tau_{\text{A}}^{2} }$$

31 $$\tau_{ \rm{max} } = \sqrt {\frac{{\sigma_{\text{A}} }}{2}^{2} + \tau_{\text{A}}^{2} }$$

where *K*_t_ and *K*_ts_ = theoretical stress concentration factors, ${\text{Mc}}$ = turning effect on the M-20 bolt, *T* = torque on the bolt and *I* = inertia of the object.

**Table 9:** Results of the stresses acting on the shoulder of the socket module of the multifunctional robotic end-effector during RVS machine maintenance

Stresses acting on the shoulder of the socket module	Value (MPa)
$\sigma_{\text{a}}$	1450.72
$T_{\text{a}}$	179.523
$\sigma_{ \rm{max} }$	1766.764
$T_{ \rm{max} }$	1041.404

Since the $\sigma_{\text{A}}$ and $\tau_{\text{A}}$ are less than $\sigma_{ \rm{max} }$ and $\sigma_{ \rm{max} }$, hence the socket design module of the multifunctional robotic end-effector is satisfactory.

### Stress simulation and analysis on the fingers and shaft of the robotic end-effector during RVS machine maintenance

The stresses acting on each of the fingers of the robotic end-effector, with thickness 20 mm each, when gripping the different RVS machine subsystems with different relative weights (obtained from [41]) were simulated on SolidWorks software. Detailed simulated stress models subjected on the fingers when grasping RVS machine subsystems are highlighted in Table 10. From Table 10, it could be affirmed that since the stresses acting on the upper arm and lower arm of the robotic end-effector finger when gripping the screen panel, torsion bar, torsion bar bracket, side plate, side liner plate, back plate, back liner plate and M-20 bolts, which are 271,331.15 and 2,713,305.3, 612,261.7 and 6,122,615.5, 196,332.8 and 1,963,323, 2,238,117 and 26,857,338, 497,017.8 and 5,964,198.5, 1,204,891.5 and 12,048,885, 451,834.35 and 4,518,332.0 and 9475.9 and 94,758.6 N/m^2^, respectively, are less than the maximum stresses at which the robotic end-effector fingers will wear out or tear apart when gripping the screen panel, torsion bar, torsion bar bracket, side plate, side liner plate, back plate, back liner plate and M-20 bolts, which are 6,511,929.5, 14,694,278, 4,711,973.5, 53,714,660, 11,928,394, 28,917,316, 10,843,994.0 and 227,420.5 N/m^2^ respectively, therefore, the stress design considerations on the fingers of the robotic end-effector during the gripping actions are satisfactory.

**Table 10:**
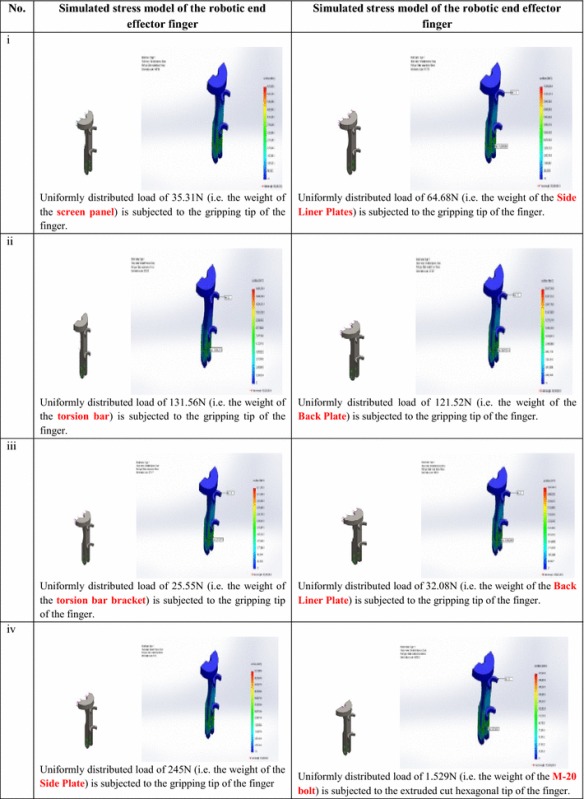
Simulated stress model results of the robotic end-effector finger during gripping actions

Detailed simulated stress models subjected on the shaft of the robotic end-effector during the gripping and ungrasping of the RVS machine subsystems are highlighted in Table 11.

**Table 11:**
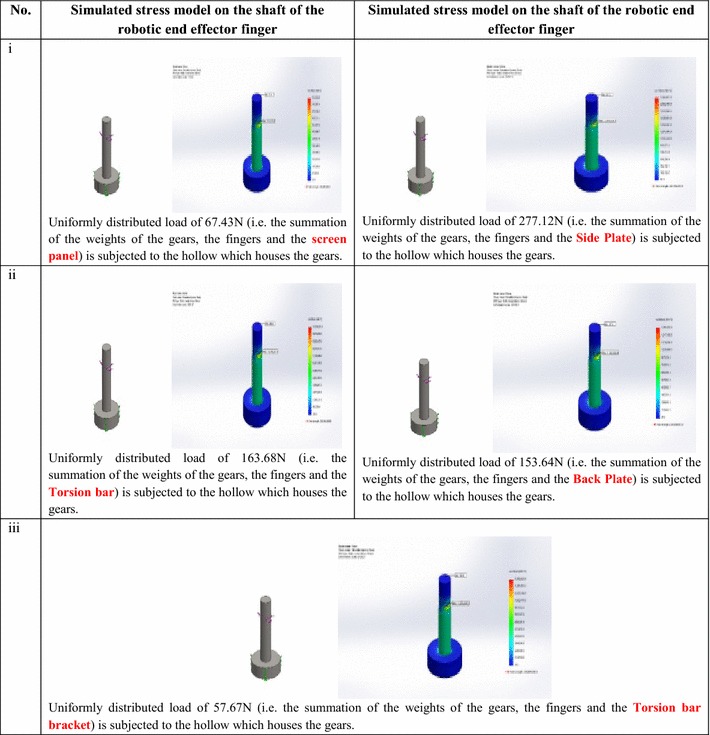
Simulated stress model results of the shaft of the robotic end-effector during gripping actions

From Table 11, it could be affirmed that since the stresses acting on the upper arm and lower arm of the shaft of the robotic end-effector when gripping the screen panel, torsion bar, torsion bar bracket, side plate and back plate, which are 33,827.8 and 473,454.7, 446,354.2 and 6,247,186.5, 57,376.8 and 803,047.6, 925,348.1 and 9,236,555.4, and 495,223.2 and 7,344,112.4 N/m^2^, respectively, are less than the maximum stresses at which the shaft of the robotic end-effector will wear out or tear apart when gripping the screen panel, torsion bar, torsion bar bracket, side plate and back plate, which are 811,620.6, 10,709,251, 1,376,625.9, 26,714,636, 181,145,528.2 N/m^2^, respectively, therefore, the stress design considerations on the shaft/lead screw of the robotic end-effector during the gripping actions are satisfactory.

### Electric cylinder actuator design analysis for hammering and unpinning task during RVS machine maintenance

**Fig. 11:**
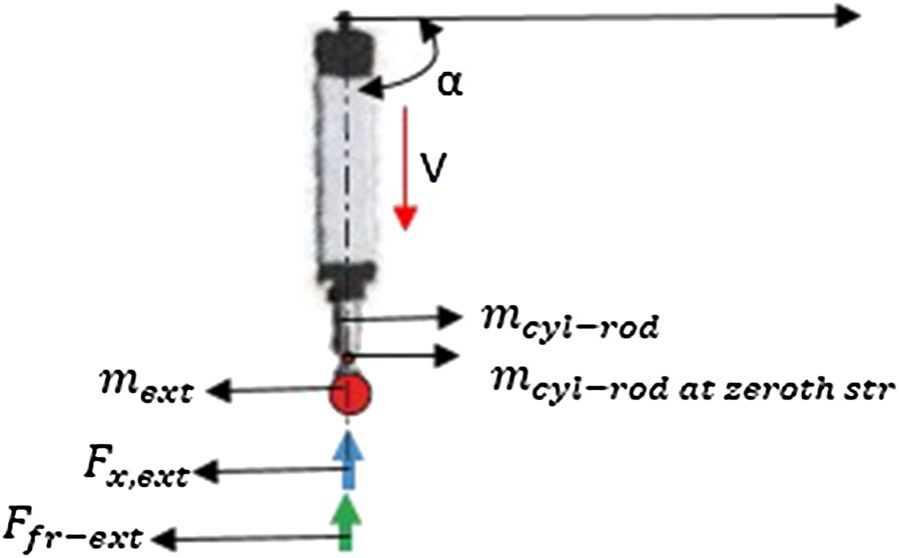
Schematic representations of the forces acting on the electric cylinder actuator module of the robotic end-effector: this image displays a simplified electric cylinder actuator module as well as forces acting on it during unpinning and hammering actions screen panel pins

Consider an electric cylinder actuator required to hammer or unpin the M-8 polyurethane screen panel pins (which hold the screen panels to the screen deck frame of the RVS machine) as depicted in Fig. 11. The relative axial forces required to hammer and unpin the screen panel pins (with a mass of 0.216 kg) from a screen panel (with mass of 3.60336 kg) during RVS machine maintenance, through the displacement of the piston rod of the electric cylinder powered by an electric motor at different operating conditions, were obtained using Eqs. (32) and (33).

32 $$F_{\text{ham}} = F_{\text{x,ext}} + F_{\text{fr-ext}} + \left\{ {m_{\text{ext}} + m_{\text{cyl-rod}} + m_{{{\text{cyl-rod}}\;{\text{at}}\; 0\;{\text{str}}}} + \left( {m_{\text{cyl-rod/mmstroke}} \times {\text{stroke}}} \right)} \right\} \times \left( {a_{\text{cyl-rod}} + g\sin \alpha } \right)$$

33 $$F_{\text{un-pin}} = F_{\text{x,ext}}-F_{\text{fr-ext}} + \left\{ {m_{\text{ext}} + m_{\text{cyl-rod}} + m_{{{\text{cyl-rod}}\;{\text{at}}\; 0\;{\text{str}}}} + \left( {m_{\text{cyl-rod/mmstroke}} \times {\text{stroke}}} \right)} \right\} \times \left( {-a_{\text{cyl-rod}} + g\sin \alpha } \right)$$

$m_{{{\text{cyl-rod}}\;{\text{at}}\; 0\;{\text{str}}}}$ = mass of the cylinder rod at zeroth stroke = 1.27 kg, $m_{\text{cyl-rod}}$ = mass of the cylinder rod = 0.27 kg, $m_{\text{cyl-rod/mmstroke}}$ = mass of the cylinder rod per mm of stroke = 7.8 kg/m, $m_{\text{ext}}$ = external mass = mass of the screen panel pin = 0.216 kg and $F_{\text{x,ext}}$ = external force = mass of the screen panel = 35.31 N.

The relative axial forces required to carry out the aforementioned tasks are highlighted in Table 12.

**Table 12:** Results of the forces required to hammer or unpin screen panel pins during RVS machine maintenance

Operating conditions of the motor powering the electric cylinder	Axial force description	Axial force values (N)
Electric motor accelerating at $4\;{\text{m/s}}^{2}$	$F_{\text{hammering}}$	33.8622
	$F_{\text{unpinning}}$	53.3658
Electric motor travelling at a constant speed of $0.63\;{\text{m/s}}$	$F_{\text{hammering}}$	25.55882
	$F_{\text{unpinning}}$	40.6896

Based on these results in Table 12, the power dissipated by the electric motor (under different operating conditions) in carrying out these hammering and unpinning actions for the aforementioned cases, which was calculated using Eqs. (34) and (35), is depicted in Table 13.

**Table 13:** Results of the power dissipated by the electric motor to hammer or unpin screen panel pins during RVS machine maintenance

Operating conditions of the motor powering the electric cylinder	Power description	Power values (W)
Electric motor accelerating at $4\;{\text{m/s}}^{2}$	$F_{\text{hammering}}$	21.33
	$F_{\text{unpinning}}$	33.62
Electric motor travelling at a constant speed of $0.63\;{\text{m/s}}$	$F_{\text{hammering}}$	16.102
	$F_{\text{unpinning}}$	25.634

34 $$P = F_{\text{ham}} \times V_{\text{m}}$$

35 $$V_{\text{m}}^{2} = U_{\text{m}}^{2} + 2as$$

On the one hand, *P* is equal to 21.33 W (i.e. 33.8622 × 0.63), when the electric motor is accelerating at 4 m/s^2^ to hammer the screen panel pins into the hollow in the screen panel or *P* is equal to 16.102 W (i.e. 25.5582 × 0.63) and when the electric motor is travelling at constant speed of 0.63 m/s to hammer the screen panel pins into the hollow in the screen panel. On the other hand, *P* is equal to 33.62 W (i.e. 53.3658 × 0.63), when the electric motor is accelerating at 4 m/s^2^ to unpin the screen panel pins from the screen panel or *P* is equal to 25.634 W (i.e. 40.6896 × 0.63) and when the electric motor is travelling at constant speed of 0.63 m/s to unpin the screen panel pins from the screen panel. Since the power dissipated for all these aforementioned cases is lower than the maximum permissible power and *P* is equal to 84,320 W (i.e. 49,600 × 1.7), at which the off-shelf electric cylinder actuator can operate, therefore the electric actuator design is safe for hammering and unpinning operation.

## Conclusion

A multifunctional robotic end-effector capable of automatically maintaining the RVS machine when used in dangerous and hazardous underground mines was sought after by the authors in this study. During these investigations, five (5) maintenance tasks of the RVS machines were analysed, which unfolded one hundred and thirty-four (134) motions required by the maintenance manager of these machines to achieve these maintenance tasks and also unveiled fifty-four (54) functions (i.e. design parameters) required in automating these maintenance tasks using the intelligent robotic solution. From these therblig and morphological results, it was inferred that the three maintenance objective functions of the robotic end-effectors required in automatically maintain the RVS machine are “griping and grasping” function (MOF1), “loosening and tightening” function (MOF2) and “unpinning and hammering” function (MOF3). Based on this confirmation, two finger modules powered by a worm and gear module capable of achieving MOF1, plug-and-play internal and external socket-inspired module attached to the tips finger modules capable of achieving MOF2 and the electric cylinder actuator module capable of achieving MOF3 were integrated together to produce a multifunctional end-effector capable of automatically maintaining the RVS machine. The detailed design evaluation of the worm–gear module (capable of ungrasping and gripping worn-out or new RVS machine subsystems), socket-inspired module (capable of loosening and tightening bolts during RVS machine maintenance) and electric cylinder actuator module (capable of unpinning and hammering screen panel pins during RVS machine maintenance) of the multifunctional robotic end-effector customized for maintaining the RVS machine were satisfactory. The multifunctional robotic end-effector designed in this study could be used in the maintenance of other mining machines such as Load–Haul–Dump (LHD) trucks, drilling rigs and crushers, which requires “gripping and ungrasping” and “loosening and bolting” functions during their maintenance operations. This research work only investigated the conceptualization, modelling and simulation of a multifunctional robotic end-effector capable of automatically maintaining the RVS machine. However, the performance evaluation of the multifunctional end-effector prototype still needs to be explored.

## Figures and Tables

**Table 14:**
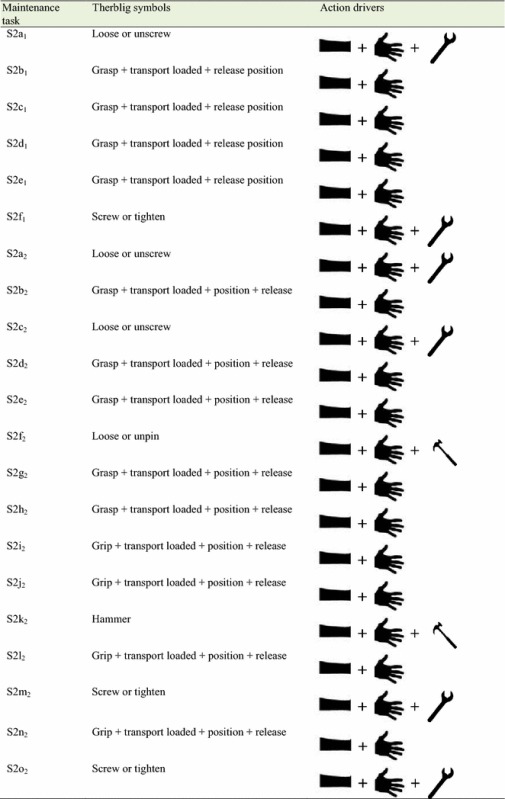
Therblig algorithm for task 2

**Table 15:**
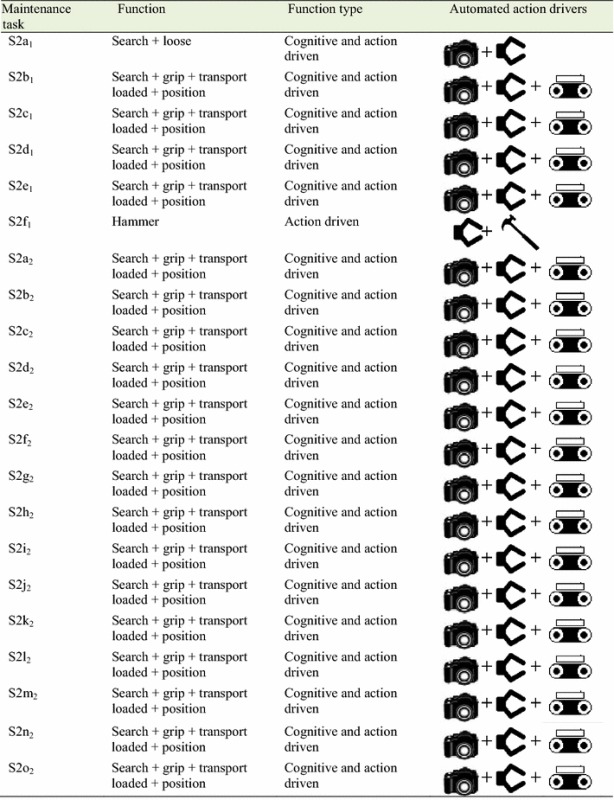
Morphological algorithm for task 2

**Table 16:**
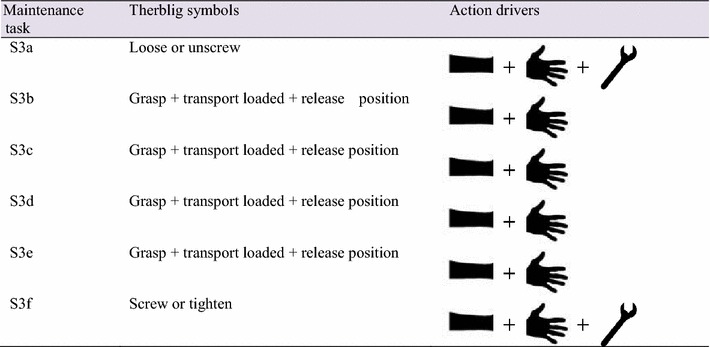
Therblig algorithm for task 3

**Table 17:**
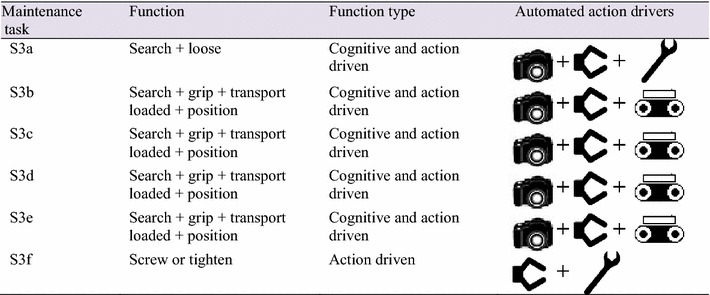
Morphological algorithm for task 3

**Table 18:**
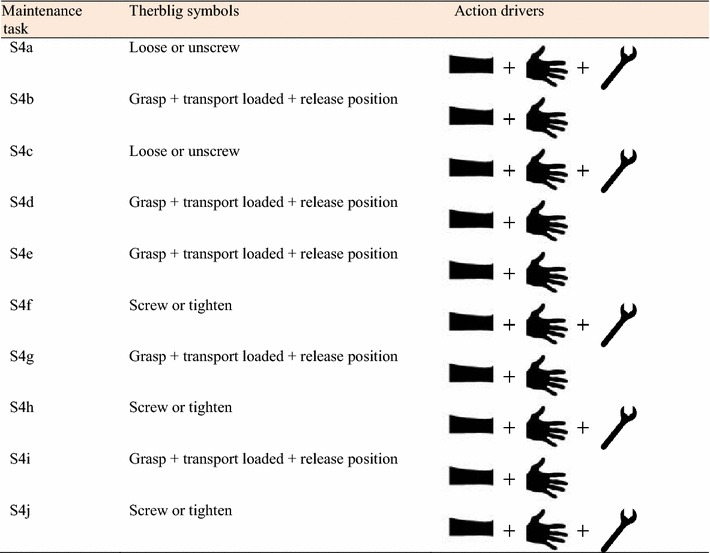
Therblig algorithm for task 4

**Table 19:**
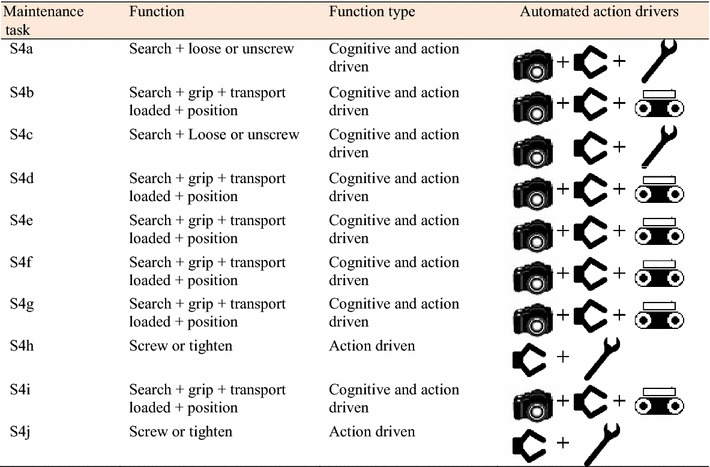
Morphological algorithm for task 4

**Table 20:**
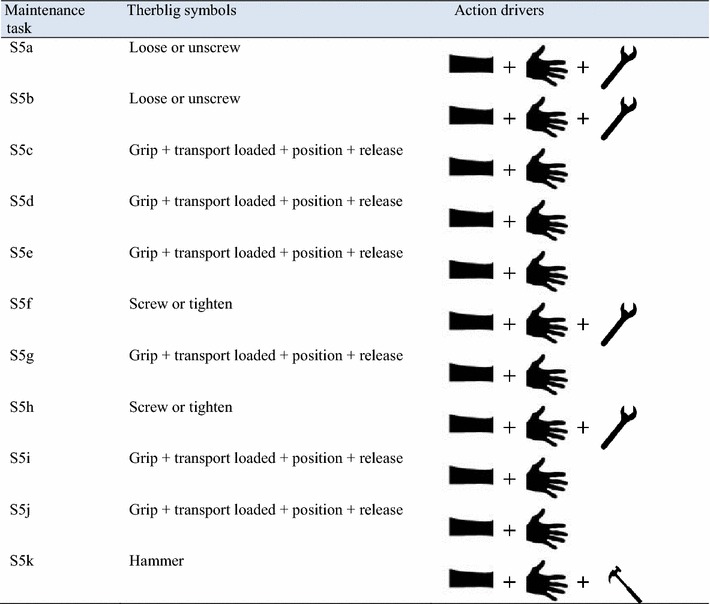
Therblig algorithm for task 5

**Table 21:**
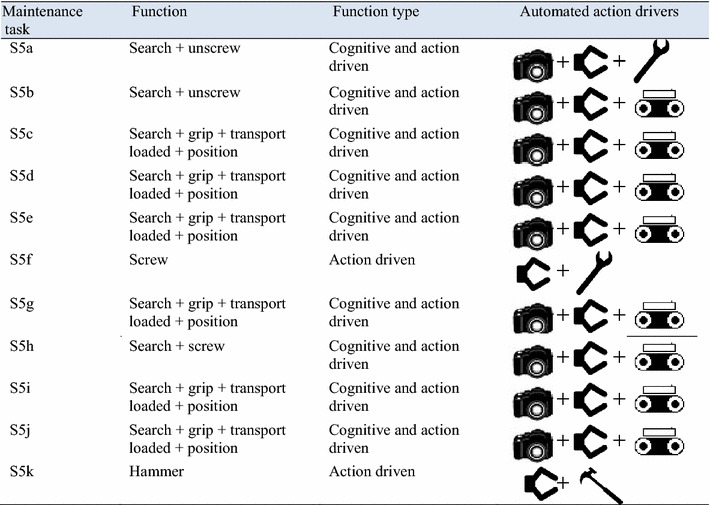
Morphological Algorithm for task 5

## Appendix 1: Description of maintenance tasks 2–5 for the RVS machine

### Task 2

- Loose the internal M16 bolts holding the worn-out side/back liner plates of the RVS machine to side plate using an Allen key bolt removal tool of the socket size capable of removing M16 bolt.
- Transport and place the internal M16 bolts either into a temporary warehouse if is still functional state condition or into the waste bin if the internal M16 bolts has worn-out.
- Grip, transport and place the worn-out side/back liner plates of the RVS machine into the waste bin.
- Grip, transport and position new side/back liner plates from the ware house where new RVS machine subsystems are stored on their exact locations on the RVS machine.
- Grip, transport and position the M16 bolts obtained either from the temporary ware house where the functional M16 bolts removed in activity (a) were kept or from the warehouse where new RVS machine subsystems are kept if the M16 bolts removed I activity (a) are worn-out on their exact location on the RVS machine.
- Tighten or screw-in the internal M16 bolts into the drilled hole made on side/back liner plate and the side/back plate using an Allen key bolt tightening tool.

However, the steps required to carry out replacement of side plate/back plate as have also worn-out then

- Loose the external M20 bolts holding the torsion bar brackets to the side plate by the means of a spanner.
- Grip, transport and place the M20 bolts into temporary warehouse.
- Loose the external M20 bolts holding the motor seat bracket to the vibrating motor.
- Transport the loose M20 bolt, loose motor seat bracket and loose vibrating motor to the temporary warehouse.
- Grip, transport and place the loose torsion bars into the temporary warehouse.
- Unpin the screen panel pins holding the screen frame to the side plate/back plate using the pin tool removal.
- Grip, transport and place the loose screen panel pins in the temporary warehouse.
- Grip, transport and place the loose worn-out side/back plate in the waste bin where worn-out subsystems are kept for recycling purposes.
- Grip, transport and place new side/back plate from the warehouse when new RVS machine subsystems are stored to their exact locations on the RVS machine.
- Grip, transport and fit the loose screen panel pins obtained from the temporary warehouse in their exact positions on the RVS machine.
- Hammer the screen panel pins into the hollow of the screen panel pins to firmly hold the new side/back liner plate to the screen deck frame.
- Grip, transport and place the loosing torsion bar brackets, loose torsion bars and the loose M20 bolts obtained from the temporary ware house in their exact positions on the RVS machine.
- Screw-in the loose M20 bolts into the drilled holes of the torsion bar bracket and tighten the torsion bar using a spanner.
- Grip, transport and place the motor bracket loose vibrating motor and M20 bolts obtained from the temporary warehouse on their exact locations on the torsion bar of the RVS machine.
- Screw-in the M20 bolts into the drilled holes of the motor seat bracket and torsion bar using a spanner.

### Task 3

- Loose the external M20 bolts holding the suspension bracket to the rosta suspension by the means of a spanner.
- Grip, transport and place the M20 bolts into temporary warehouse.
- Grip, transport and place the worn-out suspension bracket into the waste bin.
- Grip, transport and place the new suspension bracket obtained where new RVS machines subsystems are kept on the exact position on the RVS machine.
- Grip, transport and place the loose M20 bolts obtained from the temporary warehouse on their exact position of the RVS machine.
- Screw-in the loosen M20 bolts on to the drilled holes of the new suspension bracket and the rosta suspension using a spanner.

### Task 4

- Loose the external M20 bolts holding the torsion bar brackets to the side plate by the means of a spanner.
- Grip, transport and place the M20 bolts into temporary warehouse.
- Loose using a spanner the external bolts holding the torsion bar to the side plate of the RVS machine.
- Grip, transport and place the external M20 bolts in a temporary warehouse where new or functional RVS machine subsystems are placed.
- Grip, transport and place the worn-out torsion bar in the waste bin.
- Grip, transport and place the new torsion bar obtained from the warehouse where new RVS machine subsystems are kept on the exact position on the RVS machine.
- Grip, transport and place functional M20 bolts, obtained from the temporary warehouse on their exact position on the RVS machine.
- Tightening or screwing the M20 bolts into the drilled holes of the new torsion bar using a spanner in order to fasten the two RVS machine subsystems together.
- Grip, transport and place the motor seat bracket, loose vibrating motor and loose M20 bolts obtained from the temporary warehouse on their exact positions on the RVS machine.
- Screw-in or tighten the loose M20 into the drilled holes of the motor seat bracket and the torsion bar in order to fasten the two subsystems together using a spanner.

### Task 5

- Loose the external M20 bolts holding torsion bar to the side plate using a spanner.
- Loose the external bolts holding the suspension system to the RVS machine base by the means of a spanner.
- Transport, position and place the loose M20 bolts in (a) and (b) in the temporary warehouse.
- Grip, transport and position the loose side plate or suspension system from their initial positions to their exact new position of the RVS machine based on the amount of extension or reconfiguration required by the maintenance manager in order to increase the screening capability of the RVS machine.
- Grip, transport and place the loose M20 bolts (holding the suspension system to the RVS machine base) obtained from the warehouse on their new positions on the RVS machine structure.
- Screw-in and tighten the loose M20 bolts into the drilled holes of the suspension system and RVS machine base in order to fasten the two components together using a spanner.
- Grip, transport and place the loose bolts (holding the side plate to the torsion bar) obtained from the temporary warehouse on their new positions on the RVS machine
- Screw-in or tighten the loose M20 bolts into the drilled holes of the side plate and the torsion bar in order to fasten the two components together using a spanner.
- Grip, transport and place the new screen panels obtained from the warehouse where new subsystems are kept on hollow extended positions of the screen deck frame of the RVS machine.
- Grip, transport, place and fit the new screen panel pins obtained from the warehouse on their exact position on the holes of the new screen panels responsible for reconfiguration of the machine.
- Hammer the screen panel pins into the holes of the new screen panels into order to firmly fasten the pins to the new screen panels using the hammering module of the pin tool removal or hammer.

## Appendix 2(a)

See Table 14.

## Appendix 2(b)

See Table 15.

## Appendix 3(a)

See Table 16.

## Appendix 3(b)

See Table 17.

## Appendix 4(a)

See Table 18.

## Appendix 4(b)

See Table 19.

## Appendix 5(a)

See Table 20.

## Appendix 5(b)

See Table 21.
